# The fed-batch production of mannosylerythritol lipids by *Ustilago maydis* DSM 4500 from hydrophilic carbon sources

**DOI:** 10.1007/s00449-024-03084-3

**Published:** 2024-09-21

**Authors:** André D. Valkenburg, George M. Teke, Robert W. M. Pott, Eugéne van Rensburg

**Affiliations:** Department of Chemical Engineering, Stellenbosch University, Stellenbosch, 7602 South Africa

**Keywords:** Biosurfactants, Glycolipids, Mannosylerythritol lipids, *Ustilago maydis*, Hydrophilic carbon sources, Upstream production

## Abstract

Glycolipids are a class of widely studied biosurfactants with excellent applicability in cosmetic and pharmaceutical formulations. This class of biosurfactants includes mannosylerythritol lipids (MELs), which have gained particular interest due to their moisturizing and healing activity for dry and damaged human skin, arising from conditions such as eczema. Traditionally, MELs have been produced by growing certain basidiomycetous yeasts on vegetable oils. However, oils are a comparatively expensive substrate, which negatively affects the economic performance of MEL production. In addition to this, vegetable oils significantly complicate the downstream processing required to produce a product with the required purity for most applications. To address these challenges, this study investigated MEL-A production exclusively from hydrophilic carbon sources by *Ustilago maydis* DSM 4500. By implementing a fed-batch production strategy, maximum MEL-A concentration of 0.87 g/L was achieved from glucose exclusively. Also, adding micronutrients (such as MnSO_4_) to MEL-A production showed a 24.1% increase in the product titer, implying other metabolites are formed, favoring MEL production.

## Introduction

Surfactants are surface-active compounds that can reduce surface and interfacial tension between immiscible phases, making their application very important in corrosion inhibitors, detergents, wetting agents, oil recovery enhancers and pharmaceutical and cosmetic formulations [1]. As a result, surfactants represent one of the largest categories of synthetic chemicals manufactured worldwide, with their global market size projected to rise to $69.13 billion by 2032 [2]. Thus far, the global demand for surfactants has been mainly met by producing synthetic surfactants from petroleum-based, non-renewable sources. In addition to this, synthetic surfactants are often toxic to the environment and non-biodegradable [3]. Hence, global interests have shifted towards surfactants derived from biological sources, commonly referred to as biosurfactants, as alternatives to synthetic surfactants [4]. This includes microbial biosurfactants, which can be produced from renewable carbon sources by various microorganisms, including different strains of bacteria, fungi, and yeasts [5].

Glycolipids are a specific class of microbial biosurfactants which have gained significant interest due to their particularly high production yield and applicability in the cosmetic and personal care industry [6, 7]. Mannosylerythritol lipids (MELs) are a specific glycolipid biosurfactant which have gained interest due to their excellent moisturizing and reparative activity towards dry and damaged human skin cells [8, 9]. In addition to this, MELs have demonstrated selective cytotoxicity towards human skin cancer cells [10]. These properties make MELs an ideal addition to cosmetic and pharmaceutical formulations aimed at treating skin conditions, such as eczema, psoriasis, and certain types of skin cancer [11]. In addition to this, MELs have demonstrated excellent reparative activity towards damaged human hair, making them highly applicable in hair care products [12]. These properties have been the main driving force behind interest in developing microbial bioprocesses capable of producing high-purity MELs at an industrial scale.

MELs typically consist of a hydrophilic 4‐O‐β‐D‐mannopyranosyl‐D‐erythritol core, attached to a varying number of hydrophobic fatty acid chains. Four main structural variants of MELs have been distinguished based on the acylation patterns of the mannose moiety. MEL‐A represents the di‐acylated form of the compound, while MEL‐B and ‐C are mono-acylated at different positions, respectively. Finally, MEL‐D represents the completely deacylated structure of the compound [7]. MEL-A, the most hydrophobic of the MEL variants, has gained particular interest due to their improved surface-active and self-assembly properties [13–15]. It has been established that certain basidiomycetous yeasts, formerly of the genus *Pseudozyma*, and now of the genera, *Moesziomyces*, *Triodiomyces*, and *Kalmanozyma*, as well as smut fungi belonging to the genus *Ustilago*, preferentially produce MEL-A as their main structural variant, as presented in Table 1 [7, 16].

**Table 1:** Common microbial producers of MEL-A and the carbon source used during production [7]

Microbial source	Carbon source	Hydrophobic sugar moiety	Main fatty acid profile	Main structural variant	References
*Mo. antarcticus* T-34	Soybean oil	4-O-$\beta$-D-mannopyranosyl-D-erythritol	C_8:0_, C_10:0_ and C_10:1_	MEL-A and MEL-B	[17]
*Mo. aphidis* DSM 70725 or DSM 14930	Soybean oil	4-O-$\beta$-D-mannopyranosyl-$\left(1\to 4\right)$-D-meso-erythritol	C_8:0_, C_10:0_ and C_10:1_	MEL-A	[18, 19]
*T. crassus* CBS 9959	Oleic acid and glucose	4-O-$\beta$-D-mannopyranosyl-(2S,3R)-erythritol	C_14:0_, C_14:1_, C_16:0_, C_16:1_ and C_18:1_	Diastereomers of MEL-A, -B and -C	[20]
*K. fusiformata* JCM 3931 T	Soybean oil	4-O-$\beta$-D-mannopyranosyl-$\left(1\to 4\right)$-D-meso-erythritol	C_6_ and C_8_	MEL-A	[21]
*Mo. rugulosus* NBRC 10877	Soybean oil	4-O-$\beta$-D-mannopyranosyl-$\left(1\to 4\right)$-D-meso-erythritol	C_8:0_, C_10:0_, C_10:1_, C_16:0_, C_18:0_ and C_18:1_	MEL-A	[22]
*U. maydis* DSM 4500 or ATCC 1482	Sunflower oil	4-O-$\beta$-D-mannopyranosyl-D-erythritol	C_6:0_, C_14:1_ and C_16:1_	MEL-A	[23]
*Candida* sp. SY-16	Soybean oil	6-O-$\beta$-D-mannopyranosyl-$\left(1\to 4\right)$-D-meso-erythritol	C_6:0_, C_12:0_, C_14:0_ and C_14:1_	MEL-A	[24]

Until now, the production of MEL-A has only been considered from hydrophobic carbon sources, such as soybean oil and sunflower oil [7]. However, various challenges arise when using vegetable oils as the carbon source for the production of MEL-A. First, vegetable oils are a relatively expensive substrate, which negatively affects the economic viability of MEL-A production [25]. Consequently, more accessible and cost-effective carbon sources for the production of MEL-A need to be identified. On the other hand, due to the structural similarity between MELs and residual oils and fatty acids, separation processes, such as chromatography, need to be employed to produce a product with the required purity for cosmetic and pharmaceutical applications. These processes are often challenging to scale and further increase production costs [25, 26]. However, by replacing hydrophobic carbon sources with hydrophilic carbon sources, which do not contain lipids and fatty acids, the downstream processing of MELs could be significantly simplified. In this case, a high-purity MEL product could be obtained by the implementation of a single separation step, perhaps in the form of solvent extraction, and the need for more advanced downstream processes, which are normally required to remove residual lipids and fatty acids, would be voided.

*Ustilago maydis* is a corn smut fungus which has shown the ability to produce mainly MEL-A from sunflower oil [23]. However, this organism is closely related to *Sporisorium scitamineum* (formerly *Ustilago scitaminea*), a sugarcane smut fungus which has been shown to produce MELs exclusively from hydrophilic carbon sources, such as glucose, fructose, and sucrose [27–29]. Therefore, this study investigated the potential of producing MEL-A from hydrophilic carbon sources by *U. maydis* DSM 4500 with the aim of identifying a potential route towards addressing the challenges associated with MEL-A production from vegetable oils. To achieve this, the growth and production of MEL-A from various common hydrophilic carbon sources by *U. maydis* DSM 4500 were considered. The carbon sources considered included glucose, fructose, sucrose, and lactose, which were selected based on their abundance in various industrial waste streams originating from the sugar, fruit and dairy processing industries.

After the production of MEL-A from these sources was established, different factors affecting the growth and MEL-A production by *U. maydis* were investigated. Despite the importance of a suitable nitrogen source for the growth of *U. maydis*, it has been established that the expression of certain genes which are essential for the production of MELs is enhanced under nitrogen-limiting conditions [30]. Therefore, different carbon-to-nitrogen (C/N) ratios were considered to understand the relationship between nitrogen availability, growth, and MEL-A production. It has been established that certain trace elements can enhance the expression of specific genes involved in the biosynthesis of MELs. Fan et al., (2014) demonstrated that the microbial production of MEL-A by *Mo. aphidis* ZJUDM34 could be increased through the addition of Cu^2+^, Mn^2+^, and Mg^2+^, while Yang et al., (2023) observed a significant increase in the production of MELs by *Mo. aphidis* DSM 70725 when the cultures were supplemented with either Fe^2+^ or Fe^3+^. Therefore, the potential of achieving enhanced production of MEL-A from hydrophilic carbon sources by *U. maydis* through the addition of these trace elements was explored in this work. In addition to this, it has been observed that pH significantly affects the production of MELs, with certain strains of basidiomycetous yeasts performing better under neutral conditions, while other strains, including the smut fungus *S. scitamineum*, performed better under acidic conditions [33, 34]. Therefore, the effect of pH on the production of MEL-A was also investigated, as shown to have an effect on the final product titre.

After the aforementioned factors were investigated, it was observed that a low C/N ratio supported optimal growth, while a high C/N ratio supported the production of MEL-A. This indicated the potential of maximizing the production of MEL-A from hydrophilic carbon sources by implementing a fed-batch production strategy. This system would maximize biomass formation by providing a medium with a low C/N ratio during growth. Then, upon nitrogen depletion, the cultures could be supplemented with additional carbon sources to support the production of MEL-A. Although this approach has not been previously explored, it has been proven to be a viable route towards achieving significantly enhanced product titres from basidiomycetous yeasts grown on vegetable oils [35].

## Materials and methods

### Chemicals and equipment

Malt extract (for microbiology), bacteriological agar (for microbiology), yeast extract (for microbiology), D-( +)-glucose (BioReagent, purity 99%), D-(-)-fructose (BioReagent, purity 99%), D-( +)-sucrose (BioReagent, purity 99%), D-( +)-lactose (BioReagent, purity 99%), sodium nitrate (purity 99%), magnesium sulphate, monobasic potassium phosphate (purity 99%), copper (II) sulfate pentahydrate (purity 98%), manganese (II) sulfate monohydrate (purity 98%), iron (III) chloride hexahydrate (purity 98%), zinc sulfate heptahydrate (purity 98%), calcium chloride hexahydrate (purity 98%), cobalt (II) chloride hexahydrate (purity 98%), ethyl acetate (purity 99.5%), chloroform (purity 99.8%), methanol (purity 99.9%), ammonium hydroxide (ACS reagent, 28.0–30.0% NH3 basis), p-anisaldehyde (98%), acetic acid (glacial, 100%), and hydrochloric acid (purity 37%) were obtained from Sigma-Aldrich, South Africa. A pure MEL-A standard (mixture of congeners, $\ge$ 95%) was obtained from Cayman Chemical, USA.

### Culture conditions

#### Microorganisms

*U. maydis* DSM 4500 from the German Collection of Microorganisms and Cell Cultures in Braunschweig, Germany was used in this study. Stock cultures were cultivated at 30 °C for 4 days on agar medium plates consisting of malt extract (30 g/L), mycological peptone (5 g/L) and agar (15 g/L) [36]. The plates were then stored at 4 °C and renewed every 2 weeks until used for further experimentation.

#### Seed cultures

The seed cultures were prepared by inoculating stock cultures into 250 ml baffled shake flasks containing 50 ml of the synthetic medium. The synthetic medium consisted of glucose (40 g/L), NaNO_3_ (3 g/L), MgSO_4_ (0.3 g/L), KH_2_PO_4_ (0.3 g/L) and yeast extract (1 g/L). This medium was adapted from [37]. Before inoculation, the shake flasks and synthetic medium were autoclaved at 121 °C for 15 min. The seed cultures were incubated on a rotary shaker (150 rpm) at 30 °C for 3 days with an initial pH of 6.

#### Microbial growth on hydrophilic carbon sources

For the growth experiments, a synthetic medium identical to the seed cultures was used; however, this medium was supplemented with different hydrophilic carbon sources. This included glucose (40 g/L), fructose (40 g/L), sucrose (38 g/L) and lactose (38 g/L). The glucose concentration was established from literature [37]. Thereafter, fructose, sucrose and lactose were added to an equimolar carbon base. The sugarcane molasses medium consisted of sugarcane molasses (108 g/L), NaNO_3_ (3 g/L), MgSO_4_ (0.3 g/L), KH_2_PO_4_ (0.3 g/L) and yeast extract (1 g/L). The molasses, which had 0.179 g sucrose/g molasses, 0.048 g glucose/g molasses, and 0.047 g fructose/g molasses, was added to the medium at this concentration in order to achieve an initial cumulative sugar concentration of approximately 40 g/L, which was consistent with the sugar concentrations used during the pure carbon source growth tests. The growth experiments were performed by inoculating 1 ml of seed culture into 250 ml baffled shake flasks with 50 ml working volume. Before adding the seed cultures, the shake flasks and synthetic medium were autoclaved at 121 °C for 15 min. After inoculation, the cultures were incubated on a rotary shaker (150 rpm) at 30 °C and continuously monitored over a period of 4 days.

#### The effect of C/N ratio on growth and MEL-A production

Cultures were inoculated as described in Sect. "Microbial growth on hydrophilic carbon sources". However, the ratio of C/N in the growth medium was increased to 83.33 g glucose/g NaNO_3_ and 166.67 g glucose/g NaNO_3_ by increasing the glucose concentration to 50 g/L, as well as decreasing the concentration of NaNO_3_ in the medium to 0.6 g/L and 0.3 g/L, respectively [33].

#### The effect of pH on growth and MEL-A production

Cultures were inoculated as described in Sect. "Microbial growth on hydrophilic carbon sources". However, the initial pH of the growth medium was decreased to 4.0 and 2.6 by the addition of a 1 M HCl solution. The pH was then strictly controlled with a citrate–phosphate buffer system. At a pH of 4.0, the buffer consisted of citric acid (1.18 g/L) and Na_2_HPO_4_ (1.09 g/L). At a pH of 2.6, the buffer consisted of citric acid (1.71 g/L) and Na_2_HPO_4_ (0.31 g/L).

#### Fed-batch production from hydrophilic carbon sources

Cultures were inoculated as described in Sect. "Microbial growth on hydrophilic carbon sources", at a pH of 6.0 and an initial C/N ratio of 13.33 g glucose/g NaNO_3_ However, on the third day of production, the cultures were then supplemented with a 25 ml solution with a carbon source concentration of 120 g/L, 240 g/L, and 360 g/L, respectively. This increased the concentration of carbon source in the culture to 40 g/L, 80 g/L, and 120 g/L carbon source, respectively. The cultures were then incubated for another 4 days on a rotary shaker (150 rpm) at 30 °C.

#### The effect of trace elements on growth and the fed-batch production of MEL-A

To determine the effect of trace elements on the production of MELs, the fed-batch production procedure described in Sect. "Fed-batch production from hydrophilic carbon sources" was repeated with the addition of a trace element solution at varying concentrations on the first day of fermentation. The cultures had an initial pH of 6.0 and an initial C/N ratio of 13.33 g glucose/g NaNO_3._ The trace element solution consisted of CuSO_4_ (40 mg/L), MnSO_4_ (400 mg/L), FeCl_3_ (200 mg/L), ZnSO_4_ (400 mg/L), CaCl_2_ (400 mg/L) and CoCl_2_ (400 mg/L) was added to the cultures at a concentration of 0.01% (v/v), 0.1% (v/v) and 1% (v/v) [38]. After the optimal concentration of the trace element solution was identified, the trace elements were added to the culture individually to understand the effect of each individual trace element on the production of MEL-A by *U. maydis* DSM 4500.

### Separation of MEL from the liquid culture

MELs were separated from the culture using a solvent extraction technique described by Morita et al., (2009b) and Oraby et al., (2020). The liquid culture was centrifuged at 10 000 rpm for 10 min to separate the cells from the culture supernatant. An equal volume ethyl acetate was then added to the supernatant and the mixture was vortexed for 5 min to extract the MELs in the supernatant. The mixture was then centrifuged at 10 000 rpm for 10 min and the ethyl acetate fraction was collected.

A portion of the MELs was removed from the supernatant along with the biomass. After the cell pellet was washed with acidic water (pH 2) to remove residual sugars, it was extracted with ethanol at an equal volume to that of the original culture sample. The ethanol fraction was separated by centrifugation and allowed to evaporate to yield an extract. This extract was suspended twice in 4 ml ethyl acetate per gram extract. The suspension was centrifuged at 10 000 rpm for 10 min and the ethyl acetate fraction was collected. The ethyl acetate fractions were then combined and evaporated under vacuum toe yield a sticky, MEL-rich extract.

### Analytical methods

#### Quantification of biomass concentration

The concentration of biomass in the liquid cultures was determined by measuring the OD of samples taken from the cultures in a UV spectrophotometer at a wavelength of 600 nm. The cell concentration was then calculated from the calibration curve.

#### Quantification of nutrient sources

The concentration of glucose, fructose, and sucrose was measured with a Dionex Ultimate 3000 HPLC System equipped with RI detector. The system was equipped with a Biorad Aminex HPX-87H column (8 × 300 mm). The quantification was performed at a temperature of 65 °C with 5 mM H2SO4 at a flowrate of 0.6 mL.min^−1^ as the eluent.

The concentrations of the nitrates in the medium were measured with a Dionex Aquion System equipped with a suppressed conductivity detector. The system was equipped with a Dionex IonPac AS4A-SC column (4.6 × 250 mm). The quantification was performed at room temperature with a mixture of 1.8 mM sodium carbonate and 1.7 mM Bicarbonate at a flowrate of 1 mL.min^−1^ as the eluent.

#### Quantification of MEL-A

The MEL-A quantification was carried out by subjecting samples from the liquid cultures to HPLC on a silica gel column (RPSep PRX-1, 4.6 × 150 mm) with a low temperature evaporative light scattering detector using a gradient solvent program consisting of various proportions of methanol and chloroform (from 100:0 to 0:100, v/v) at a flow rate of 1 mL.min^−1^. The pure sample of MEL-A, obtained from Cayman Chemicals, was used as a standard.

#### Thin-layer chromatography

The purified MEL extract was dissolved in ethyl acetate at a concentration of 50 g/L and dropped onto a silica TLC plate (Gel 60 F254, Merck) and before being developed in a solvent mixture of chloroform, methanol and ammonium hydroxide at a volumetric ratio of 65:15:2. An anisaldehyde solution was then prepared by adding 0.5 mL of anisaldehyde in 10 mL of glacial acetic acid. To this solution were added 85 mL of methanol and 5 ml of concentrated sulfuric acid, in that order. The TLC plates were then lightly sprayed with the anisaldehyde solution and heated to 110 °C for 5 min. MELs appeared as purple spots on the developed TLC plates.

## Results and discussion

This section presents a demonstration of the production of MEL-A from hydrophilic carbon sources (mono- and disaccharide sugars) using *U. maydis* DSM 4500 with the aim of addressing the challenges associated with the production of MELs from vegetable oils. Different operating conditions were investigated in order to maximize the product titres achieved from hydrophilic carbon sources, including the C/N ratio and pH of the medium. Fed-batch production was implemented to further increase the titre of MEL-A which could be achieved without vegetable oils. In addition to this, the most important trace elements affecting MEL-A production by *U. maydis* DSM 4500 were identified.

### Production of MEL-A from hydrophilic carbon sources

#### Biomass growth on hydrophilic carbon sources

**Fig. 1:**
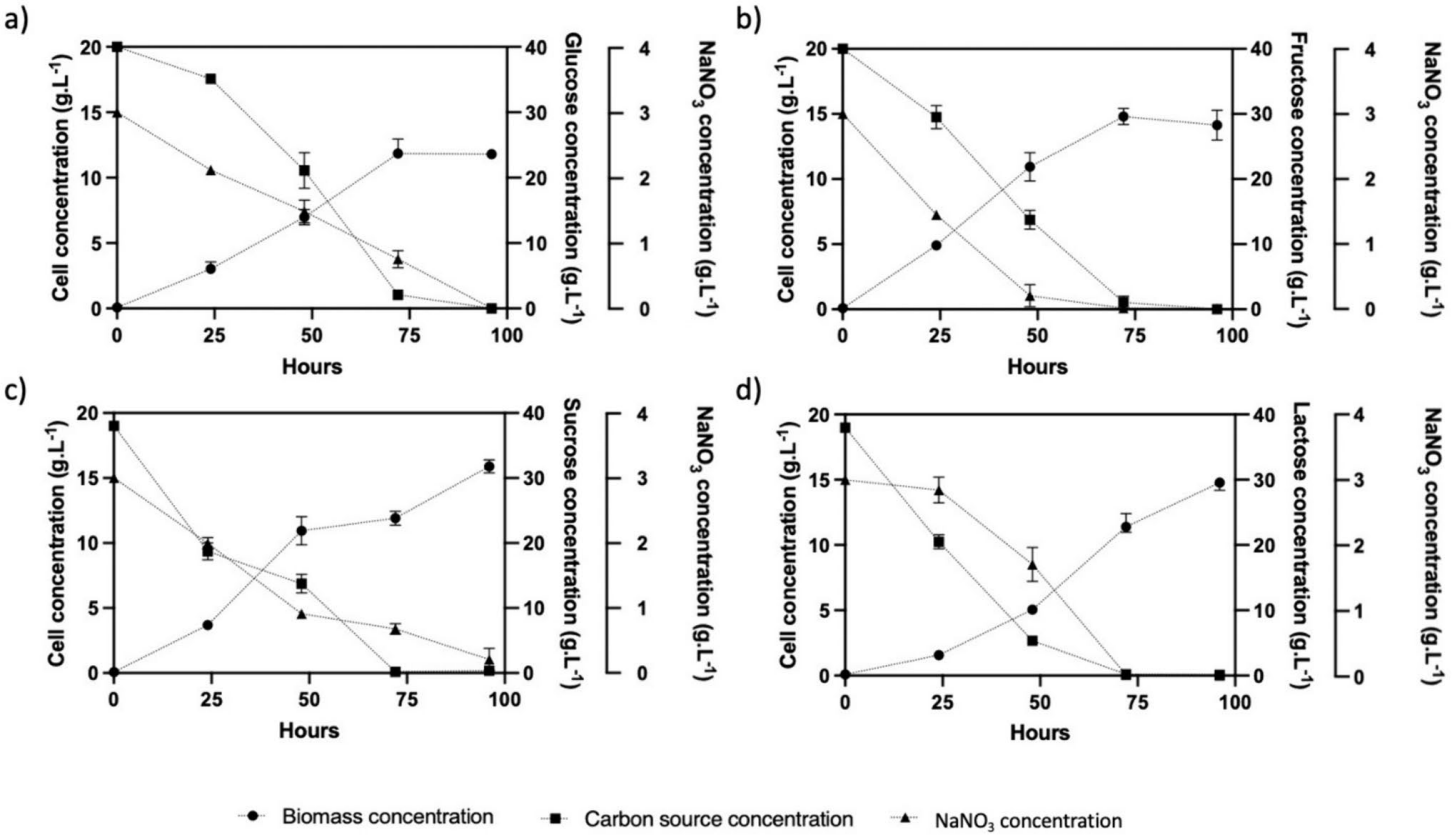
The growth and substrate consumption of * U. maydis* DSM 4500 cultivated on different carbon sources. The carbon sources were **a** glucose at a concentration of 40 g/L, **b** fructose at a concentration of 40 g/L, **c** sucrose at a concentration of 38 g/L and **d** lactose at a concentration of 38 g/L. The error bars represent the standard deviation of triplicate replicates

The growth of *U. maydis* DSM 4500 on four hydrophilic carbon sources (glucose, fructose, sucrose, and lactose) is shown in Fig. 1. The monosaccharide carbon sources, glucose and fructose resulted in the highest biomass growth rates, with the organisms reaching stationary phase after 72 h and achieved maximum biomass concentrations of 14.84 g/L and 12.2 g/L with fructose and glucose, respectively. This represented biomass yields per gram carbon consumed of 0.36 g/g and 0.305 g/g on fructose and glucose, respectively. Furthermore, during the growth stage, the organism achieved a growth rate of 0.201 g/L.h and 0.169 g/L.h on fructose and glucose, respectively. Similar results were obtained by Liebal et al., (2022) who consistently achieved biomass yields per gram substrate consumed between 0.3 g/g and 0.4 g/g over a large range of initial glucose concentrations. Furthermore, Liebal et al., (2022) observed a growth rate of 0.18 g/L.h when *U. maydis* MB215 was grown on glucose at an initial concentration of 50 g/L.

Despite showing slightly decreased growth rates of 0.165 and 0.153 g/L.h on sucrose and lactose, respectively, comparable biomass concentrations of 15.88 g/L and 14.66 g/L were achieved when stationary phase was reached on day 4.

Furthermore, the utilization of the disaccharides led to an increased yield of biomass per gram substrate, as shown in Fig. 3. These results demonstrated the potential of growing *U. maydis* DSM 4500 on a wide range of feedstocks consisting of different mono- and disaccharide sugars. The decreased growth rate on the disaccharides was attributed to the additional time required for the organism to hydrolyze the disaccharides into their monosaccharide constituents. Sucrose was hydrolyzed into glucose and fructose, while lactose was hydrolyzed into glucose.

**Fig. 2:**
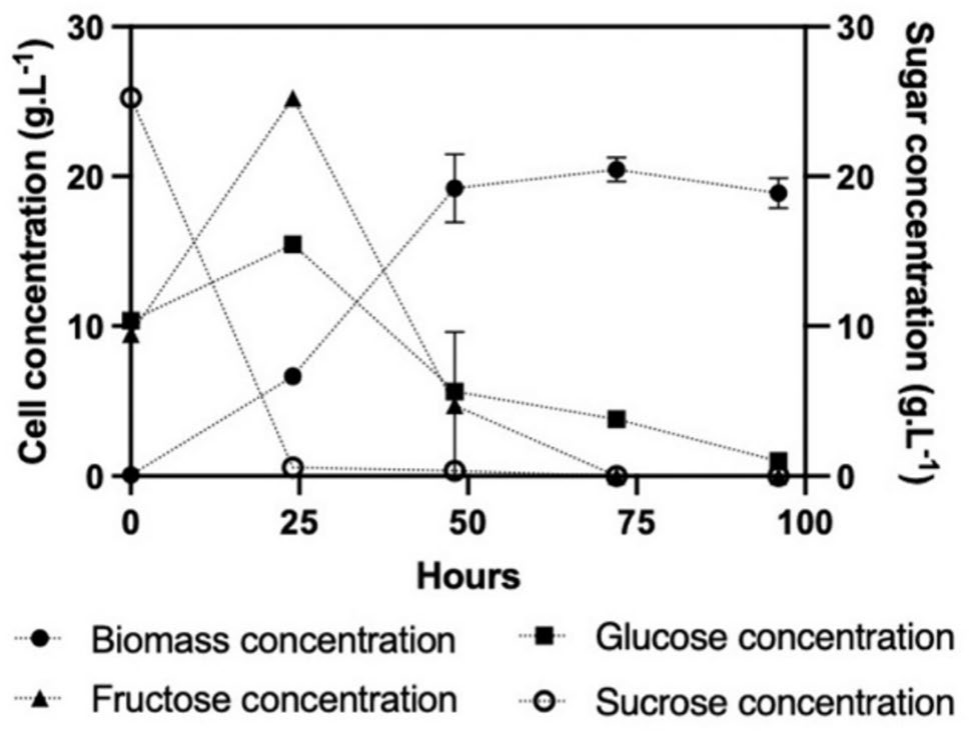
Growth and substrate consumption of * U. maydis* DSM 4500 cultivated on sugarcane molasses as the sole carbon source. The error bars represent the standard deviation of triplicate replicates

The hydrolysis of sucrose into glucose and fructose was demonstrated when *U. maydis* DSM 4500 was grown on sugarcane molasses, as shown in Interestingly, the concentration of fructose increased significantly more than glucose concentration during this period, indicating that the organism preferentially utilized the glucose during the early growth stage, which was released after invertase activity, thus permitting continuous growth of the organism until the glucose and fructose were depleted. However, after the first day, fructose was depleted at a faster rate when compared to glucose. This could have simply been due to the higher concentration of fructose present in the medium. However, this was likely also affected by more complex metabolic factors which would warrant further investigation, as shown in Fig. 2.

**Fig. 3:**
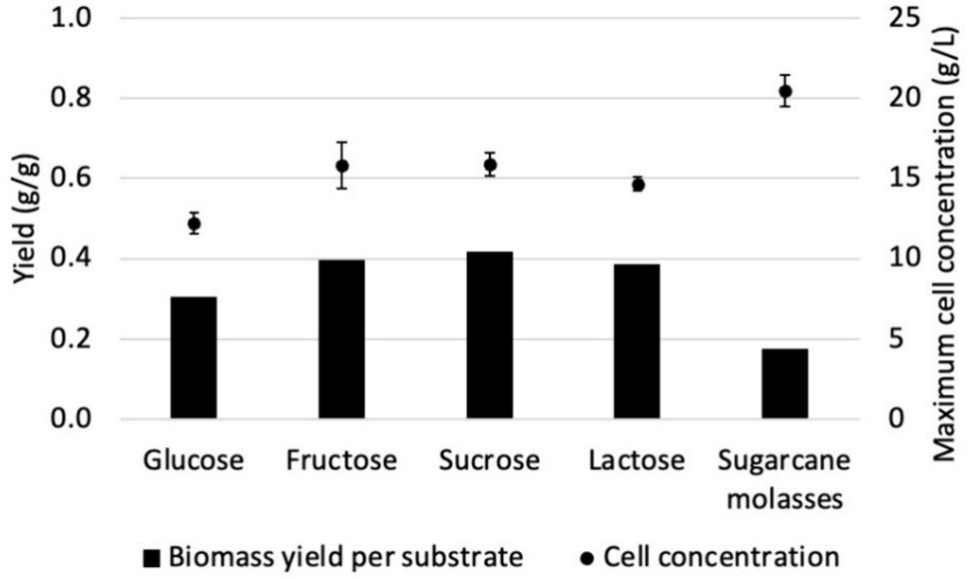
Comparing the growth of * U. maydis* DSM 4500 after 96 h on pure hydrophilic carbon sources to the that achieved on sugarcane molasses. The error bars represent the standard deviation of triplicate replicates

Despite achieving an increased biomass concentration on sugarcane molasses in comparison to the pure mono-and disaccharide sugars, the utilization of sugarcane molasses led to a significantly decreased biomass yield per gram substrate consumed, as shown in Fig. 3. However, considering the significant decreased cost of sugarcane molasses in comparison to technical grade sugar feedstocks, this decrease in biomass yield could potentially be justified if significant production could be achieved.

Sugarcane molasses is a common industrial waste stream with high concentrations of sucrose, fructose, and glucose, which has been identified as a cost-effective and renewable substrate for microbial bioprocesses. When grown on sugarcane molasses, it was observed that all of the sucrose was depleted in the first 24 h, while the concentration of both glucose and fructose increased. Interestingly, the concentration of fructose increased significantly more than glucose concentration during this period, indicating that the organism preferentially utilized the glucose during the early growth stage, which was released after invertase activity, thus permitting continuous growth of the organism until the glucose and fructose were depleted. However, after the first day, fructose was depleted at a faster rate when compared to glucose. This could have simply been due to the higher concentration of fructose present in the medium. However, this was likely also affected by more complex metabolic factors which would warrant further investigation.

Despite achieving an increased biomass concentration on sugarcane molasses in comparison to the pure mono-and disaccharide sugars, the utilization of sugarcane molasses led to a significantly decreased biomass yield per gram substrate consumed, as shown in Fig. 3. However, considering the significant decreased cost of sugarcane molasses in comparison to technical grade sugar feedstocks, this decrease in biomass yield could potentially be justified if significant production could be achieved.

In terms of product formation, no MEL-A production was observed after 96 h of growing *U. maydis* DSM 4500 on the mono- and disaccharide sugars*.* After performing an analysis on the genes involved in the biosynthesis of MELs by *U. maydis*, Hewald et al., (2006) established that MEL production is induced under nitrogen limitation. However, as shown in Fig. 1, when nitrogen depletion was achieved, the carbon source was also depleted. This implied that the lack of MEL-A production could be attributed to the lack of carbon availability for product formation after nitrogen limitation was achieved. To verify this, the C/N ratio in the medium was increased in order to ensure the availability of carbon upon nitrogen depletion with the aim of inducing the production of MEL-A.

#### The effect of C/N ratio on the production of MEL-A

The production of MEL-A was investigated at various ratios of glucose to NaNO_3_ (13.33 g glucose/g NaNO_3_, 83.33 g glucose/g NaNO_3_ and 166.67 g glucose/g NaNO_3_).

**Fig. 4:**
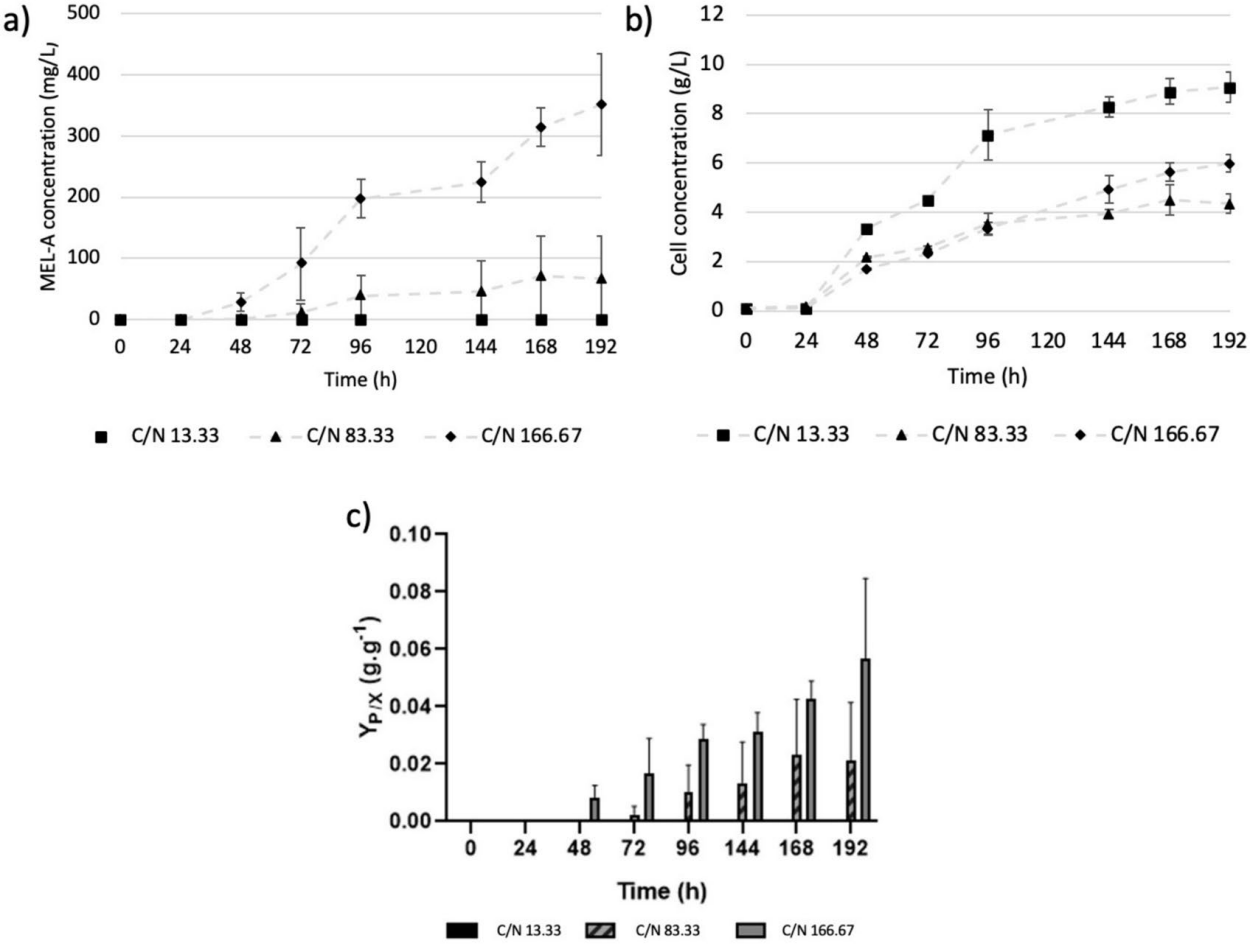
**a**) The production of MEL-A by *U. maydis* DSM 4500 at varying C/N rations. **b** The gram biomass produced by *U. maydis* DSM 4500 at varying C/N rations. **c** The MEL-A produced per gram *U. maydis* DSM 4500 biomass produced at varying C/N rations. YP/X refers to the gram product produced per gram biomass produced. The error bars represent the standard deviation* of triplicate replicates *

As shown in Fig. 4a, increasing the C/N ratio to 83.33 g glucose/g NaNO_3_ and 166.67 g glucose/g NaNO_3_, led to final MEL product titres of 0.067 g/L and 0.351 g/L, respectively, demonstrating that the presence of a hydrophilic carbon source upon nitrogen depletion induced the production of MELs.

As shown in Fig. 4a, MEL-A formation commenced after 50 h, when stationary phase and nitrogen depletion were achieved, demonstrating that the production of MEL-A by *U. maydis* DSM 4500 is not associated with the growth of the organism and is highly dependent on nitrogen starvation.

It was observed that the ratio of C/N in the growth medium affects the production of MEL-A in two ways. First, as shown in Fig. 4b, increasing the C/N in the growth medium decreased the biomass concentrations achieved at stationary phase. This decreased the amount of resting cells which could produce MEL-A.

Alternatively, as shown in Fig. 4c, the yield of MEL-A per gram biomass produced increased when the C/N ratio was increased, indicating that resting cells could more effectively produce MEL-A under higher C/N ratios. These findings indicate the potential of implementing a fed-batch production strategy which is initially focused on maximizing biomass formation during the growth phase in the presence of nitrogen, before shifting focus to the production of MEL-A during the stationary phase by supplementing the cultures with additional carbon source upon nitrogen depletion.

#### The effect of pH on the production of MEL-A

**Fig. 5:**
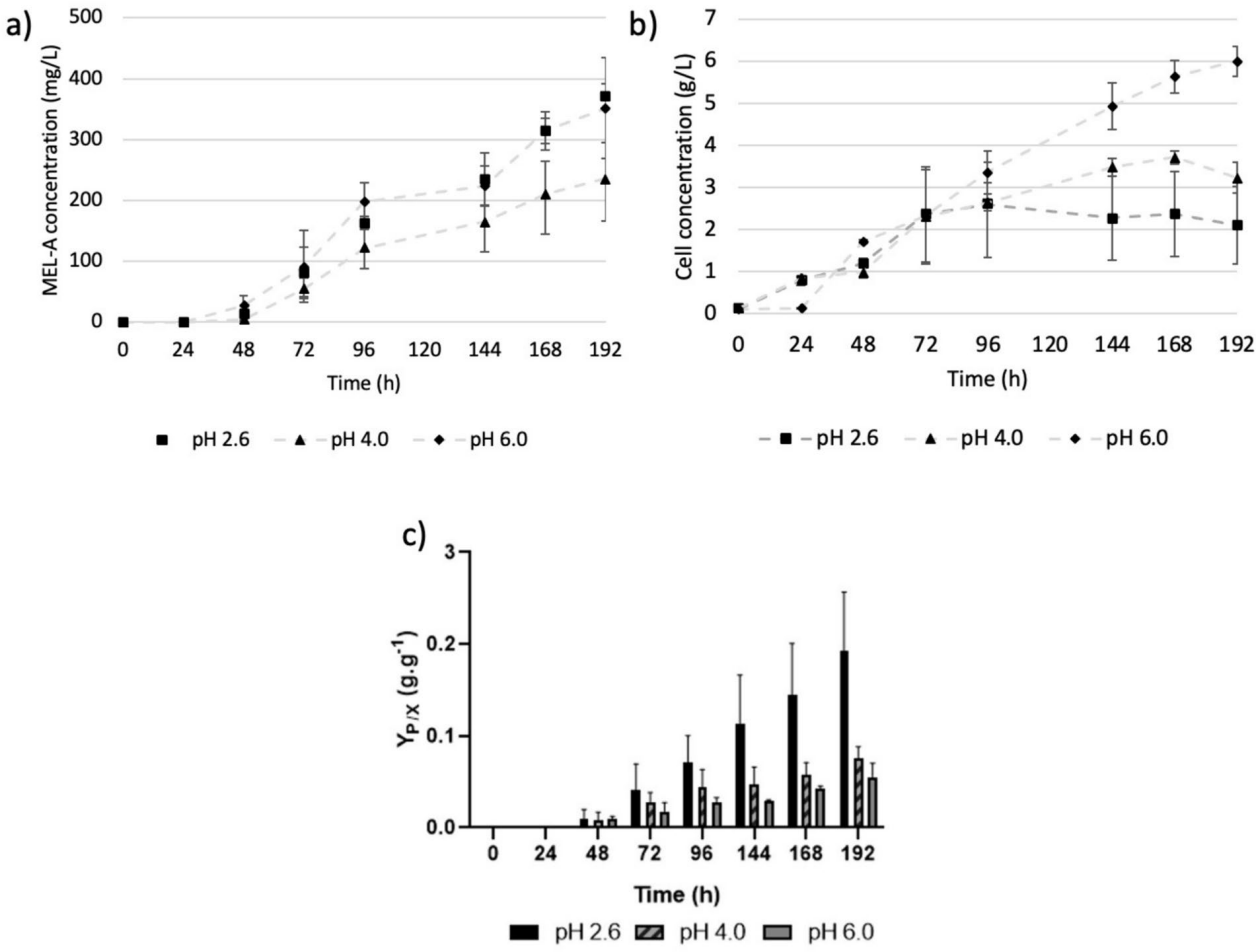
**a** The production of MEL-A by * U. maydis *DSM 4500 at a pH of 2.6, 4.0, and 6.0. **b** The gram biomass produced by *U. maydis* DSM 4500 at a pH of 2.6, 4.0, and 6.0. **c** The gram MEL-A produced per gram * U. maydis *DSM 4500 biomass produced at a pH of 2.6, 4.0 and 6.0. Y_P/X_ refers to the gram product produced per gram biomass produced. The error bars represent the standard deviation of triplicate replicates

The pH of the production medium is another important variable affecting the production of glycolipids by smut fungi of the family *Ustilaginaceae* [33]. Therefore, in order to investigate the effect of decreasing the pH of the medium on the production of MEL-A by *U. maydis* DSM 4500, the fermentation was performed at a pH of 6.0, 4.0, and 2.6, with a glucose to NaNO_3_ ratio of 166.67 g glucose/g NaNO_3_. As shown in Fig. 5a and b, final MEL-A titres of 0.351 g/L, 0.236 g/L and 0.371 g/L were achieved at a pH of 6.0, 4.0 and 2.6, respectively. Therefore, a clear trend between the pH of the culture and the production of MEL-A could not be established. As shown in Fig. 5b**,** final biomass titers of 6.68 g/L, 3.24 g/L and 2.10 g/L were achieved at a pH of 6.0, 4.0 and 2.6, respectively, indicating that the organism grew less effectively under increasingly acidic conditions. This was further shown by an increased concentration of unconsumed glucose in the medium under increasingly acidic conditions, with a final glucose concentration of 3.41 g/L, 39.17 g/L, and 46.59 g/L being measured at a pH of 6.0, 4.0 and 2.6, respectively. These findings correlated with the biomass concentrations achieved at each pH.

When considering the grams of MEL-A produced per gram biomass, shown in Fig. 5c, a clear trend was observed. In this case, it was observed that the product yield per biomass increased with a decreasing culture pH. These results demonstrated that, similar to the ratio of C/N in the growth medium, the pH of the growth medium affects the production of MEL-A in two ways. First, biomass formation is decreased under more acidic conditions, leading to a decreased number of viable cells which can produce MEL-A. On the other hand, when considering the increase in the product yield per biomass under increasingly acidic conditions, it is clear that the biomass could produce MEL-A more effectively at a lower culture pH. This explains why the product titers, presented in Fig. 5a, could be maximized at a pH of either 2.6 or 6.0. Furthermore, the lowest product titers were achieved at a pH of 4.0 since the system favored neither biomass formation nor MEL-A production. These results demonstrated the potential of implementing more advanced production protocols in which the pH is optimized for biomass formation during the growth stage, before the pH is decreased upon the onset of nitrogen depletion to maximize the production of MEL-A.

### The fed-batch production of MEL-A

**Fig. 6:**
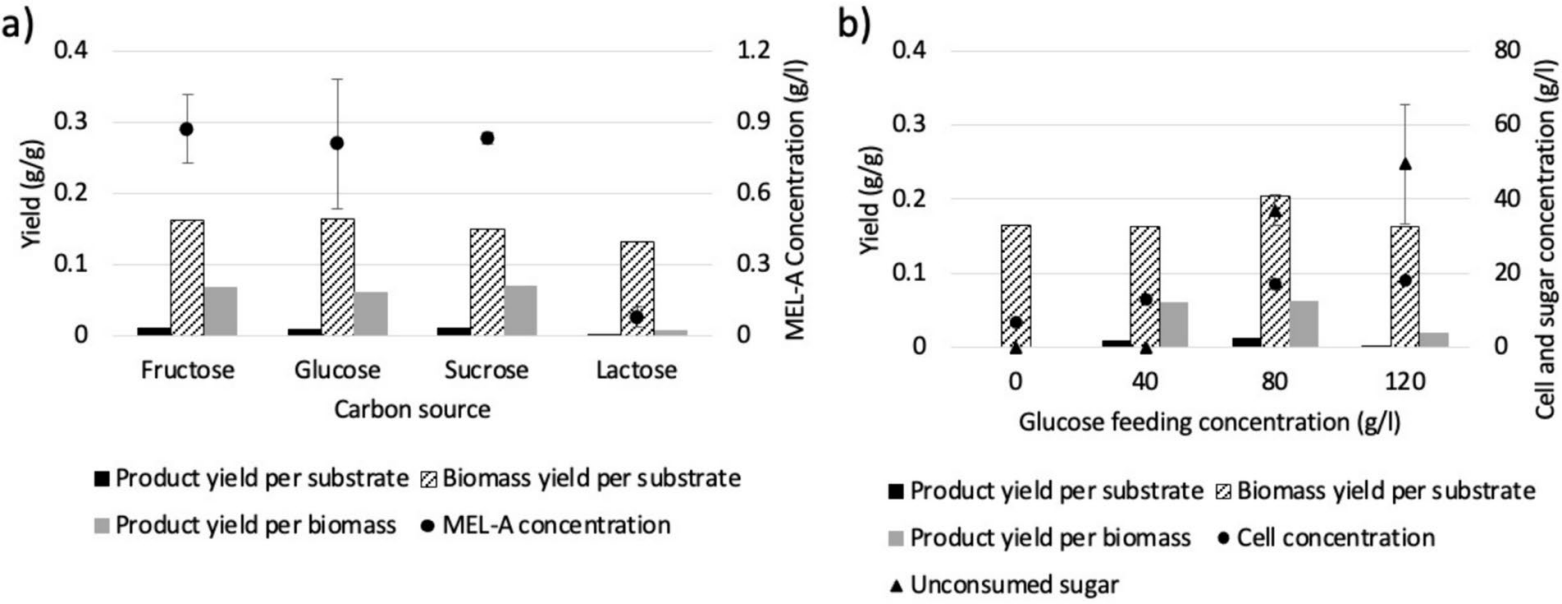
**a** The product titres and yields achieved by the fed-batch production of MEL-A by * U. maydis* DSM 4500 by feeding 40 g/l of different hydrophilic carbon sources. **b** Investigating performance of the fed-batch production of MEL-A by * U. maydis* DSM 4500 with different concentrations of glucose feeding on the 3rd day. The error bars represent the standard deviation of triplicate replicates

In the fed-batch production strategy, the production of MEL-A with the supplementation of 40 g/L glucose, fructose, sucrose, and lactose on the third day of fermentation was considered. As shown in Fig. 6a, the production of MEL-A could be significantly increased through the supplementation of additional carbon source upon nitrogen depletion. Similar product titres of 0.87 g/L, 0.81 g/L and 0.83 g/L could be achieved from glucose, fructose and sucrose, respectively, after 7 days of fermentation. These results indicated the potential of utilizing industrial waste streams with high concentrations of glucose, fructose, and sucrose for the production of MEL-A by *U. maydis* DSM 4500. This includes waste streams from the sugar processing industry, such as sugarcane bagasse and molasses, as well as waste from the fruit industry. On the other hand, lactose proved to be a less suitable carbon source for the production of MEL-A, achieving a final product titre of 0.08 g/L after 7 days. This suggests industrial waste streams which originate from the dairy industry are not suitable for the production of MEL-A by *U. maydis* DSM 4500.

As shown in Fig. 6b, final MEL-A titres of 0.87 g/L, 1.07 g/L and 0.38 g/L were achieved after 7 days when the cultures were supplemented with an additional 40 g/L, 80 g/L and 120 g/L of glucose on the 3rd day, respectively. This represented product yields 0.01, 0.013 and 0.003 g MEL-A produced per grams glucose consumed and production rates of 0.116, 0.153 and 0.054 g/L per day, respectively. When the cultures were fed with 40 g/L and 80 g/L glucose, there was not a significant difference it in the achieved product yields per substrate or production rates. However, this was likely due to the high concentration of unconsumed glucose (37.16 g/L) which remained in the growth medium of the culture supplemented with 80 g/L fructose after 7 days of production. On the contrary, the carbon source was completely depleted after 7 days in the culture which was supplemented with 40 g/L glucose. Therefore, the MEL-A titre in the culture supplemented with 80 g/L glucose would likely have been significantly increased if the production period was increased.

As shown in Fig. 6a and b, a significant decline in the growth, product titre, product yields per substrate, and production rate was observed when the cultures were supplemented with 120 g/L glucose on the 3rd day of production. The decreased efficiency in the growth of *U. maydis* DSM 4500 was likely due to damage caused to the cells by the high hydrostatic pressures associated with the high concentration of glucose in the growth medium. Furthermore, the decreased product titre and yield were likely due to the strain favoring the production of trehalose over MEL-A due to the high osmotic pressure. Salmerón-Santiago et al., (2011) showed that *U. maydis* ATCC 201384 FB2 accumulated the disaccharide trehalose intracellularly in response to extreme environmental changes, including osmotic stress and temperature. Similar results were obtained by Cervantes-Chávez et al., (2016), who observed that a mutant strain of *U. maydis*, in which a gene which is essential for trehalose production, trehalose-6-phosphate phosphatase (Tps2), was removed, showed increased sensitivity towards various stresses, including oxidative, heat, acid, ionic and osmotic stresses. Although the presence of trehalose was not verified, it was clear that a feeding the cultures with high concentration glucose solutions inhibited the strain’s ability to effectively produce MELs.

#### The effect of different trace elements on the fed-batch production of MEL-A

**Fig. 7:**
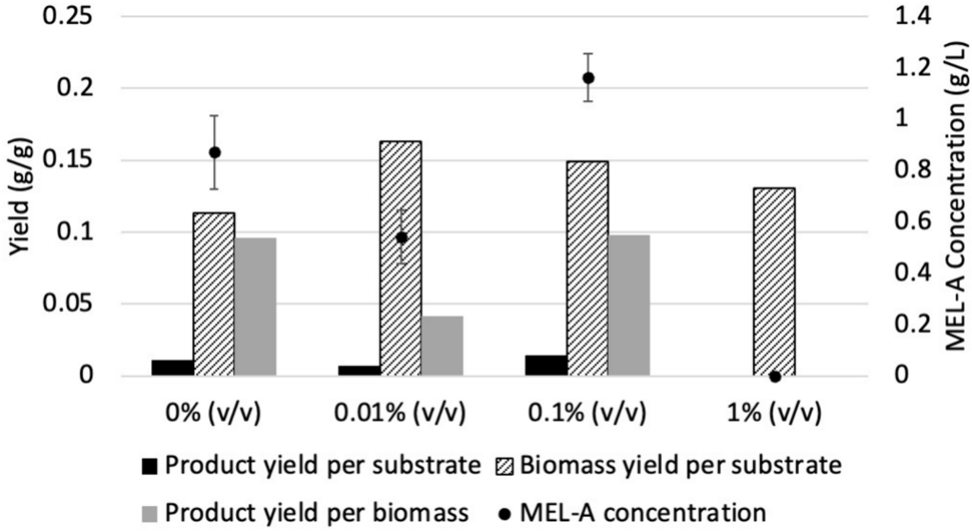
The effect of adding a defined trace element solution at varying concentrations on the fed-batch production of MEL-A from glucose by * U. maydis *DSM 4500. The error bars represent the standard deviation of triplicate replicates

The effect of different trace elements on the production of MEL-A was investigated with the aim of achieving enhanced production. As shown in Fig. 7, when the cultures were not supplemented with additional trace elements, the final MEL-A and biomass concentrations were 0.87 g/L and 9.05 g/L, respectively. When the cultures were supplemented with the trace element solution at a concentration of 0.1% (v/v), the final MEL-A and biomass concentrations could be increased to 1.16 g/L and 11.91 g/L, respectively. Finally, adding the trace element solution at a concentration of 1% (v/v) reduced the production of MEL-A to a concentration of 0.037 g/L, with a final cell concentration of 10.47 g/L. This indicated that certain trace elements were toxic to *U. maydis* at higher concentrations.

**Fig. 8:**
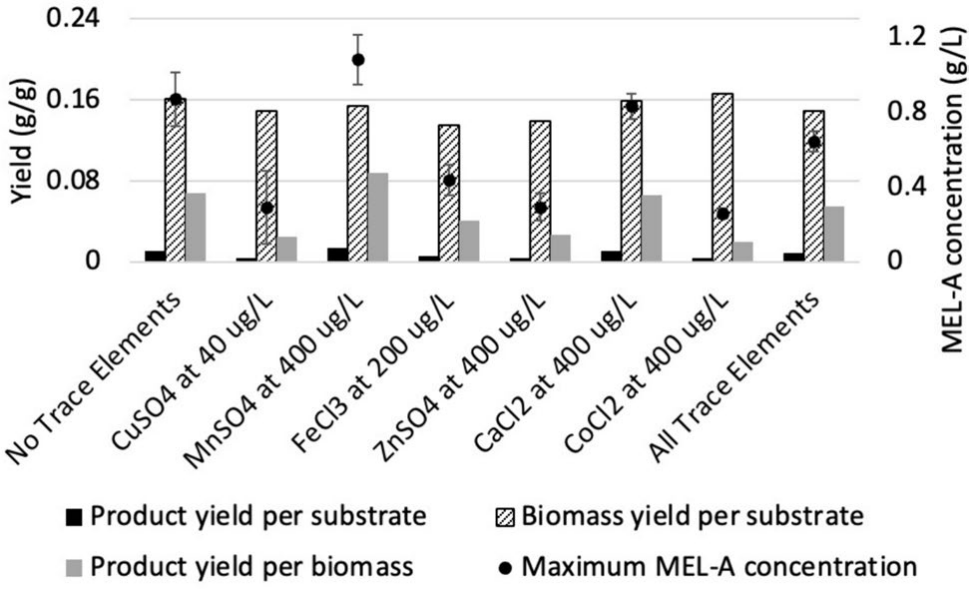
The effect of different trace elements on the fed-batch production of MEL-A from glucose by * U. maydis* DSM 4500. Y_P/S_ refers to the gram product produced per gram substrate consumed, Y_P/X_ refers to the gram product produced per gram biomass produced, Y_X/S_ refers to the gram biomass produced per gram substrate consumed. The error bars represent the standard deviation of triplicate replicates

When the trace elements were added to the cultures individually, as shown in Fig. 8, it was observed that the addition of Mn^2+^, in the form of MnSO_4_, enhanced the production of MEL-A to a final product titre of 1.08 g/L. This represented a 24.1% increase in product formation in comparison to the fed-batch production of MEL-A from glucose without supplementation of any trace elements. Similar results were obtained by Fan et al., (2014), who observed that the production of MEL-A by *P. aphidis* ZJUDM34 was enhanced by supplementing the culture with Mn^2+^. Supplementing the cultures with Ca^2+^, in the form of CaCl_2_, and Fe^3+^, in the form of FeCl_3_, led to final product titres of 0.799 g/L and 0.442 g/L, respectively. Yang et al., (2023) observed a similar decrease in the production of MEL-A when a *P. aphidis* DSM 70725 culture was supplemented with Ca^2+^ at a concentration of 1 mM. However, Yang et al., (2023) observed a significant increase in the production of MEL-A by 17% and 32% by supplementing the *P. aphidis* DSM 70725 cultures with Fe^2+^ and Fe^3+^, respectively. This indicated that, although Fe^2+^ and Fe^3+^ play an important role in the biosynthetic pathway for the production of MEL-A, their presence at these concentrations had an inhibitory effect on both the growth of *U. maydis* DSM 4500 and the subsequent production of MEL-A. The addition of CuSO_4_, ZnSO_4_, and CoCl_2_ had significant inhibitory effects on the production of MEL-A without dramatically affecting the biomass yield per gram substrate consumed.

Therefore, it is thought that the addition of these trace elements shifts production towards the formation of other metabolites in favor of MEL-A, such as itaconic acid or cellobiose lipids [42]. However, the production of these metabolites was not investigated here.

## Conclusions

It was observed that *U. maydis* DSM 4500 can be efficiently grown to high cell concentrations on various mono- and disaccharide sugars. Consequently, it was shown that *U. maydis* DSM 4500 can be effectively grown to high biomass concentrations on sugarcane molasses. It was shown that the production of MEL-A increased when the C/N ratio was increased to 83.33 g glucose/g NaNO_3_ and 166.67 g glucose/g NaNO_3_, respectively. However, a C/N ratio of 13.33 g glucose/g NaNO_3_ led to significantly higher biomass production. During the growth phase, biomass formation is maximized through the availability of nitrogen. Thereafter, the organism is supplemented with additional carbon source upon nitrogen starvation to maximize the production of MEL-A. It was observed that pH affects MEL-A production in two ways. First, biomass formation is favored under more neutral conditions, increasing the concentration of viable cells capable of producing MEL-A. Alternatively, the product yield per biomass is enhanced when the culture pH decreases, indicating that the cells can more effectively produce MEL-A under more acidic conditions. Maximum MEL-A concentrations of 0.87 g/L, 0.81 g/L and 0.83 g/L could be achieved by supplementing the organisms with 40 g/L of glucose, fructose and sucrose, respectively. When the cultures were supplemented with 0.4 mg/L MnSO_4_, the product titre could be increased to 1.08 g/L, corresponding to a product yield per glucose consumed of 0.013 g/g, and a production rate of 0.0064 g/L/d, representing the maximum titre and yields achieved in this study. It was found that supplementing the cultures with 0.4 mg/L CaCl_2_ and 0.2 mg/L FeCl_3_ also enhanced the production of MEL-A. Although the utilization of hydrophilic carbon sources facilitated the downstream processing of MELs, the product yields achieved under these conditions were not comparable to those achieved from hydrophobic carbon sources. However, this study serves as a proof of concept for the production of MELs exclusively from hydrophilic carbon sources. Nevertheless, further research is required in order to further optimize the titres achieved from these carbon sources, as well as to identify if the techno-economic performance of MEL production is improved by simplifying the downstream processes with the omittance of hydrophobic carbon sources during fermentation.

## Data Availability

Data will be made available on request.
